# Comparison of outcomes between vertical and transverse skin incisions in percutaneous tracheostomy for critically ill patients: a retrospective cohort study

**DOI:** 10.1186/s13054-018-2174-y

**Published:** 2018-09-30

**Authors:** Sung Yoon Lim, Won Gun Kwack, Youlim Kim, Yeon Joo Lee, Jong Sun Park, Ho Il Yoon, Jae Ho Lee, Choon-Taek Lee, Young-Jae Cho

**Affiliations:** Division of Pulmonary and Critical Care Medicine, Department of Internal Medicine, Seoul National University College of Medicine, Seoul National University Bundang Hospital, 82, Gumi-ro 173 Beon-gil, Bundang-gu, Seongnam-si, Gyeonggi-do 13620 Republic of Korea

**Keywords:** Percutaneous tracheostomy, Vertical skin incision, Transverse skin incision, Tracheostomy site ulcers

## Abstract

**Background:**

Percutaneous tracheostomy (PT) is a common procedure in critical care medicine. No definite clinical practice guidelines recommended on the choice of the direction of skin incision, vertical or transverse for tracheostomy in critically ill patients. The objective of this retrospective study was to compare the outcomes associated with vertical and transverse skin incisions in patients undergoing PT.

**Methods:**

Patients who underwent PT between March 2011 and December 2015 in the intensive care unit (ICU) of a tertiary hospital were retrospectively included. PTs were performed by pulmonary intensivists at the ICU bedside using the single tapered dilator technique assisted by flexible bronchoscopy. The primary outcome was the incidence of tracheostomy site ulcers at 7 days after PT.

**Results:**

Of the 458 patients who underwent PT, a vertical incision was made in 27.1% and a transverse incision was made in 72.9%. There were no tracheostomy-related mortalities, and no significant difference in the incidence of immediate postoperative complications, including bleeding, tracheal ring fracture, and subcutaneous emphysema. Thirty-five patients (7.6%) developed complications within 7 days after PT, in which tracheostomy-related pressure ulcers were the most frequent. Compared with vertical incisions, transverse incisions were associated with significantly lower incidence of complications (14.1% vs. 5.4%, *P* = 0.001).

**Conclusions:**

This retrospective study showed that transverse skin incisions in PTs for critically ill patients, resulted in a significant decrease in overall complications, particularly ulcers in the tracheostomy site.

## Background

Tracheostomy is one of the most frequently performed procedures in critically ill patients requiring mechanical ventilation. Since the initial report of the dilatational technique by Ciaglia in 1985 [1], percutaneous tracheostomy (PT) has largely replaced conventional surgical tracheostomy (ST) in intensive care unit (ICU) patients. The most commonly cited advantages are the ease of performance and reduced complications compared to ST. As it can be safely performed at the bedside and avoids moving the patient to the operating room, PT is now considered as a viable alternative to ST in the ICU settings [2–4].

Since the initial description of the dilatational technique by Ciaglia [1], vertical skin incision has been traditionally used in PT. A vertical skin incision could be extended further downward to perform tracheostomy, if necessary at any time, and is less likely to injure lateral neck vasculature and other adjacent structures. Contrarily, surgeons typically use a transverse skin incision in ST, and a vertical skin incision has only been used in case of an emergency, as this can be implemented quickly [5]. In addition, a transverse skin incision allows for improved healing and more esthetic outcomes than do conventional incisions. However, there is little evidence on which incision type is the best choice for PT in critically ill patients. In this retrospective study, we aim to compare the outcomes associated with the two skin incision types, i.e., transverse and vertical incisions, in critically ill patients undergoing PT in the ICU.

## Methods

**Fig. 1:**
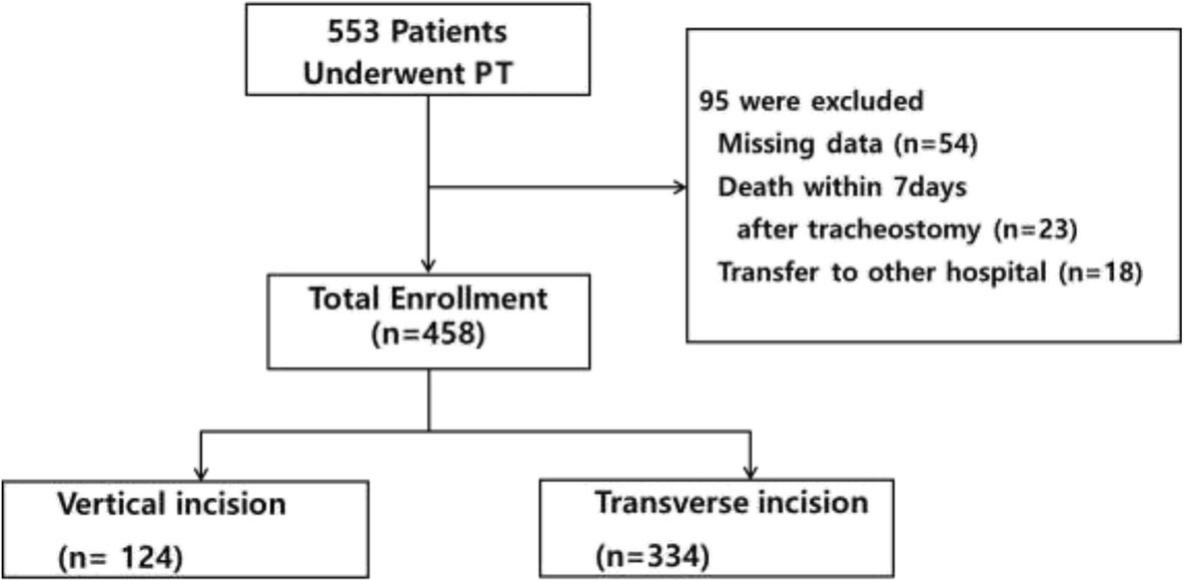
Diagram showing the flow of participants through each stage of the study

This retrospective cohort study was approved by the Institutional Review Board of Seoul National University Bundang academic hospital (approval number B-1709-423-102). All patients aged 18 years or older who underwent their first PT in the ICU of our hospital between 1 March 2011 and 31 December 2015 were included. The exclusion criteria were as follows: (1) patients without accurate records of postoperative outcomes, (2) patients who died within 7 days after tracheostomy without any procedural complication related to the procedure, and (3) patients transferred to other facilities within 7 days after tracheostomy (Fig. 1).

The patients’ demographics and clinical characteristics, including indication for tracheostomy, date of tracheostomy, perioperative complication, length of hospital stay, and in-hospital mortality, were collected and reviewed. We analyzed the complications that developed within the first 48 h postoperatively, including subcutaneous emphysema, obstruction, and false passage or dislodgement of the tube, which were defined as perioperative complications. The primary outcome was the incidence of tracheostomy site ulcers at 7 days after PT. Secondary outcomes were complications within 7 days after PT, including bleeding, hematoma, and cellulitis at the tracheostomy site, with reference to a prior study.

### Surgical technique

All PTs were performed by medical intensivists or a fellow (under supervision) in our hospital, using the single tapered dilator technique (Ciaglia Blue Rhino Percutaneous Tracheotomy Introducer Kit; Cook Critical Care™, Bloomington, IL, USA). Under fiberoptic bronchoscopic guidance, the PT procedure was performed as follows. After appropriate patient positioning and skin preparation, a skin incision was made about 1 cm below the cricoid cartilage either vertically or transversally per operator’s decision. The correct placement of the tracheostomy tube was achieved by palpation of standard anatomical landmarks and through bronchoscopic assistance. Ultrasound scanning was used if the tracheal anatomy was not readily palpable in a physical examination. The introducer needle was advanced through the incision and into the trachea. Once proper placement was confirmed, a guide wire was advanced. The needle was then removed while leaving the guide wire in place. A series of dilatations were performed over the guide wire. Once dilatation was deemed satisfactory, the tracheostomy tube was then inserted over the guide wire or the dilator. The duration of the procedure (from skin incision to tracheostomy tube placement) and operative complications, if any, were noted after tracheostomy tube placement [6–8].

### Post tracheostomy care

**Fig. 2:**
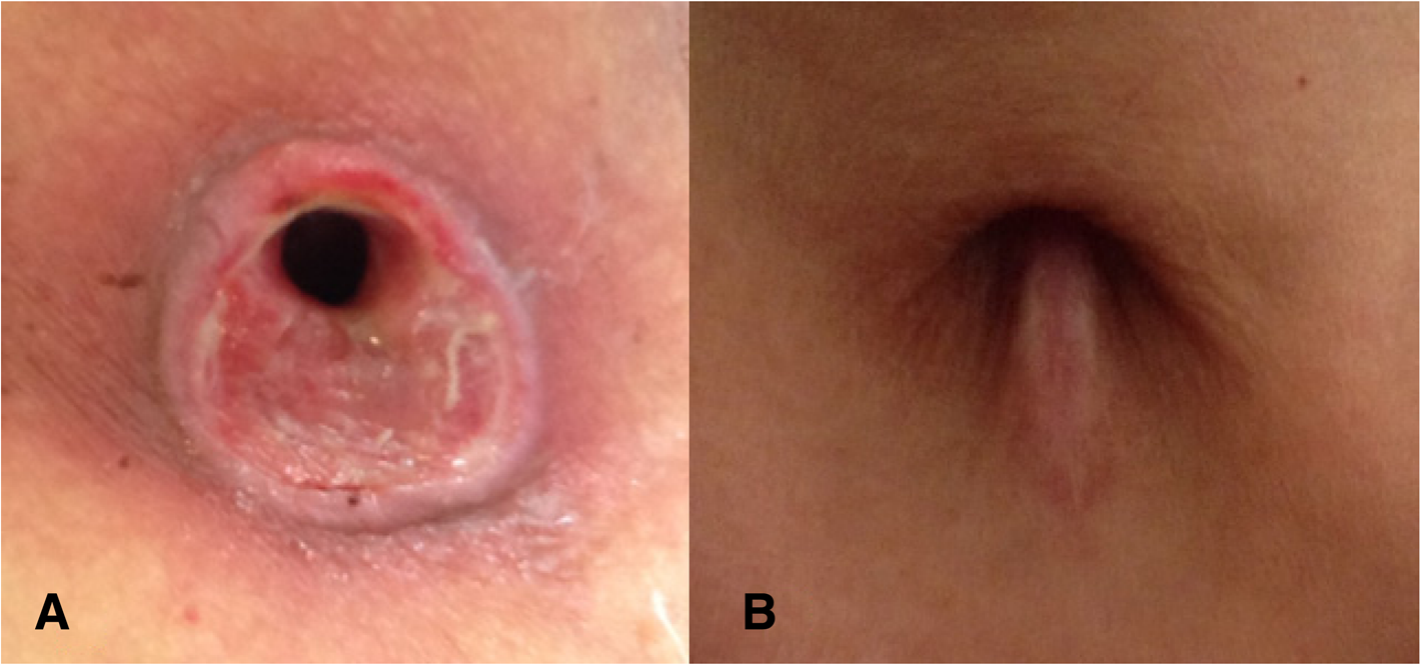
**a** Stomal site ulcer after vertical incision. **b** Healed ulcer

We applied absorbent foam dressing under the tracheostomy tube in the immediate postoperative period as routine practice to reduce tracheostomy wound complications. The medical intensivist who performed or supervised the PT procedure routinely assessed the tracheostomy wounds 3 and 7 days postoperatively and documented the wound status in the patients’ medical records, per our institution’s post PT care protocol. The bedside ICU nurses also vigilantly monitored the wounds, and they took photos of wounds and notified the operator if necessary (Fig. 2a and b). In cases of uncontrolled bleeding and delayed wound healing, we consulted the otolaryngologist for surgical interventions. The first change of the tracheostomy tube was usually around 7 days postoperatively.

### Statistical analysis

Comparisons between the two groups were performed with the Mann–Whitney U test for numerical data, and the χ2 test or Fisher’s exact test for categorical data. To identify the risk factors for PT-related complications, variables that were statistically significant (*P* < 0.05) in the univariate analysis were then included in the multivariate logistic regression analysis with forward conditional elimination of data. Data are presented as odds ratios (ORs) with 95% confidence intervals (CIs). A two-tailed *P*-value <0.05 was considered to indicate statistical significance. SPSS software (version 19.0; IBM, USA) was used for statistical analyses.

## Results

A total of 458 patients were included in this study (Table 1). The patients’ mean age at time of PT was 68 years, and the study cohort predominantly comprised men. Patients were grouped according to the skin incision type, i.e., vertical or transverse skin incision; 124 (27.1%) underwent tracheostomy with a vertical skin incision, and 334 (72.9%) underwent tracheostomy with a transverse incision. No significant differences were noted in demographics and severity score on the Acute Physiology and Chronic Health Evaluation (APACHE) II system (25.98 vs. 26.23, respectively) between the group that underwent tracheostomy with a vertical skin incision and the group that underwent tracheostomy with a transverse incision. The most common reasons for performing a PT were need for long-term airway maintenance and need for prolonged mechanical ventilation. Under mechanical ventilation support, the mean day on which tracheostomies were performed was day 10 from ICU admission in both groups. The proportion of patients taking antiplatelet agents did not differ between the vertical and transverse skin incision groups; however, the proportion of patients taking anticoagulants was higher in the vertical skin incision group than in the transverse skin incision group (14.7 vs 8.7%, *P* = 0.039). The percentage of attending physicians performing PT was similar for the two groups.

**Table 1:** Baseline characteristics of the patients who underwent PT

Characteristics	Vertical (*n* = 124)	Transverse (*n* = 334)	*P* value
Age, years	68.4 ± 15.1	68.2 ± 15.7	0.948
Sex, male (%)	77 (60.7)	223 (66.8)	0.553
BMI	20.5 ± 4.9	20.7 ± 3.7	0.294
Duration of mechanical ventilation before PT (day)	10.6 ± 6.3	9.7 ± 7.7	0.437
APACHE II	25.98 ± 6.5	26.23 ± 8.3	0.567
Reason for tracheostomy (%)			0.211
Need for long-term free airway maintenance	73 (58.8)	178 (53.2)	
Prolonged mechanical ventilation	50 (40.3)	140 (42.0)	
Laboratory findings (coagulation)			
Platelets (10^3^/μl)	207	208	0.695
PT (INR)	1.12	1.15	0.229
aPTT (s)	40.15	39.60	0.988
Antiplatelet agent during perioperative period (%)	17 (13.6)	36 (10.8)	0.472
Aspirin	14 (11.2)	22 (6.6)	
Clopidogrel	2 (1.6)	5 (1.5)	
Aspirin and clopidogrel	1 (0.8)	9 (2.7)	
Anticoagulant during perioperative period (%)	18 (14.7)	29 (8.7)	0.039
Operated on by attending physician (%)	82 (65.3)	198 (59.3)	0.190
Periprocedural parameters			
Size of incision (cm)	1.4 ± 0.2	1.3 ± 0.2	0.046
Procedure time (min)	19.3 ± 9.0	16.10 ± 8.8	< 0.005
Length of stay in ICU	25.4	24.0	0.001
Length of stay in hospital	61.6	74.8	0.067
In-hospital mortality	30 (24)	70 (20.8)	0.494

Values are presented as number (%) or mean ± SD

*APACHE* Acute Physiology and Chronic Health Evaluation, *BMI* body mass index, *PT* percutaneous tracheostomy, *PT INR* the international normalized ratio of prothrombin time, *aPTT* activated partial thromboplastin time

The procedural success rate was 100%, and there was no case of conversion to ST. None of the patients developed periprocedural life-threatening complications. Although the operation time was marginally longer in the vertical incision group, there was no significant difference in the incidence of immediate postoperative complications, including bleeding, tracheal ring fracture, and subcutaneous emphysema (Tables 1 and 2). The most frequent perioperative complication was bleeding (vertical versus transverse, 9.7 vs. 4.5%; *P* = 0.036). Additionally, there was no significant difference in the duration of hospital stay and ICU stay and the rate of in-hospital mortality between the two groups. Mortality was not related to tracheostomy in either group.

**Table 2:** Postoperative complications in the two groups

Characteristics	Vertical (*n* = 124)	Transverse (*n* = 334)	*P* value
Immediate postoperative complications	24 (19.3)	41 (12.3)	0.083
Unintended extubation	2 (1.6)	3 (0.9)	0.513
Bleeding			0.036
Minor (3–5 small, soaked swabs)	12 (9.7)	15 (4.5)	
Major	0 (0)	0 (0)	
Tracheal ring fracture visible by bronchoscopy	7 (5.6)	18 (5.4)	0.915
Desaturation (≤ 88% during procedure)	1 (0.8)	0 (0)	0.100
Subcutaneous emphysema	1 (0.8)	2 (0.6)	0.807
Others^a^	1 (0.8)	3 (0.9)	0.925
Complications developed within 7 days after PT	17 (14.1)	18 (5.4)	0.001
Delayed bleeding	2 (1.8)	1 (0.3)	0.122
Hematoma	0 (0)	1 (0.3)	0.542
Tracheostomy site ulcer	8 (6.5)	4 (1.2)	0.002
Cellulitis	3 (2.4)	4 (1.2)	0.344
Others (erythema)	4 (3.2)	8 (2.4)	0.621
Long-term complications	2 (1.6)	4 (1.2)	0.728
Tracheal stenosis	2 (1.6)	2 (0.6)	0.358
Tracheomalacia	0 (0)	2 (0.6)	0.388

Values are presented as number (%)

*PT* percutaneous tracheostomy

^a^Two patients had hypotension and two had tachycardia

Of 35 patients (7.6%) who developed complications within 7 days after PT, the vertical incision group developed more common complications than the transverse group (14.1% vs. 5.4%; *P* = 0.001; Table 2). Notably, among the complications associated with PT, ulcers at the tracheostomy site were noted in 6.5% of patients in the vertical incision group, and in only 1.2% of patients in the transverse incision group. In multivariate analysis, vertical skin incision was found to be an independent risk factor for ulcers at the tracheostomy site (odds 2.821; CI 1.327–5.996; *P* = 0.007; Table 3).

**Table 3:** Risk factors for tracheostomy site ulcer identified by multiple logistic regression analyses

	Univariate analysis	*P* value	Multivariate analysis	*P* value
	Odds ratio (95% CI)		Odds ratio (95% CI)	
Age (years)	1.004 (0.979–1.029)	0.760		
Male sex	1.288 (0.599–2.771)	0.517		
Antiplatelet agent during perioperative period	1.624 (0.668–3.949)	0.285		
Anticoagulant agent during perioperative period	1.239 (0.467–3.289)	0.667		
APACHE II	0.968 (0.920–1.018)	0.200		
Duration of MV before PT	0.977 (0.922–1.034)	0.415		
Size of incision	0.560 (0.171–1.832)	0.337		
Procedure time	0.999 (0.999–1.000)	0.092	0.999 (0.999–1.000)	0.038
Operated on by attending physician	0.734 (0.431–1.250)	0.255		
Vertical (versus transverse)	3.014 (1.387–6.554)	0.005	2.821 (1.327–5.996)	0.007
Immediate postoperative complications	1.133 (0.481–2.668)	0.775		

*APACHE* Acute Physiology and Chronic Health Evaluation, *PT* percutaneous tracheostomy, *MV* mechanical ventilator, *CI* confidence interval

## Discussion

In the present study, transverse skin incisions were associated with lower incidence of complications occurring within 7 days postoperatively compared to vertical skin incisions. Ulcers at the tracheostomy site, which was one of the most common complications noted in our study, was predominantly observed in the vertical skin incision group. Critically ill patients who require PT frequently have multiple risk factors for development of pressure ulcers, including malnutrition, sepsis, anemia, immobility, and neurologic injuries. In this study, we found that many of the patients requiring PT did have comorbidities, but none of these were significantly related to skin breakdown around the tracheostomy wound site.

Ulcers at the tracheostomy site are mostly pressure ulcers caused by the tracheostomy tube creating a constant pressure over the skin of the incision site, with additional disruption of skin integrity due to wetness from respiratory secretions [9]. When the tracheostomy tube is in direct contact with the incision wound, and exerts a force caused by both gravity and friction, it results in angulation and stretching of blood vessels within the sub-dermal tissues, which in turn causes thrombosis and cellular death. This consequently manifests as necrosis and undermining of the deepest layers [10]. A vertical skin incision might be under more gravitational pressure from the tracheostomy tube and ventilator circuit, and thus it is more susceptible to pressure ulcers than a transverse skin incision.

Not only gravitational force, but hampered blood supply to the skin in vertical incision might affect the increased incidence of pressure ulcer. Rabson et al. reported that the blood supply of the neck skin anterior to the trapezius muscle descends from the facial and occipital arteries and ascends from the transverse cervical artery [11]. However, the midline of the neck skin where the incision was made is supplied from the branches of these major vessels running in a horizontal direction [12]. Due to the anatomical nature of the horizontal branching vessels, vertical incision of the anterior neck skin would comprise the cutaneous blood supply and tend to offer increased risk of development of pressure ulcer. However, we were not able to conclude exactly which mechanisms contribute to frequent development of tracheostomy site ulcer in patients with a vertical incision. Further studies are required to clarify the underlying mechanisms.

Ulcers at the tracheostomy site are commonly acquired complications in pediatric patients. There are no published prevalence rates of pressure ulcers caused specifically by PT, but several studies have reported that tracheostomy wounds complicate pediatric tracheotomy at a rate of 8–19% [9, 13]. In our retrospective study, the rate of skin breakdown or pressure ulcer formation after PT in adult patients was 3.3%; this is less often than in the pediatric population. There are factors specific to pediatric populations that increase the risk of pressure ulcers on the skin around a tracheostomy tube, such as inability to verbally express pain, the presence of moisture from expelled tracheal secretions, and more fragile skin [14–16].

There were no significant differences in perioperative complications between the vertical and transverse skin incision groups. Bleeding caused by injury to the superficial neck veins present underneath the skin incision made during PT was likely a main problematic complication, because most of the PTs were performed without a suture. The vertical skin incision made during PT was expected to reduce the risk of vascular injuries, excessive bleeding, and possibly, conversion to open tracheostomy, but the incidence of bleeding as a complication was not different between the groups, and there was no case of conversion to open tracheostomy in our study. The average reported rate of bleeding as a complication is approximately 11% [17], which is comparable to the finding of our study. Paradoxically, lower incidence of bleeding as a complication in the transverse skin incision group was possibly due to a preoperative decision to avoid transverse incision in patients with prominent anterior jugular veins.

In patients with skin-related complications in our study, standard wound care was administered by intensivists and bedside nurses, and there were very few patients needing surgical revision or developing esthetic problems. However, no standardized staging system for skin breakdown around the tracheotomy wound site was routinely utilized in this study. Improper wound classification could lead to underestimating or underreporting of tracheostomy wound complications.

In surgical or open tracheostomy for an acute airway emergency, a vertical skin incision may be required for rapid access to the trachea. However, the vertical incision should not be used routinely as it is a cosmetically unappealing direction of skin incision resulting in a more esthetic outcome than transverse incisions. As this was a retrospective study primarily depending on the third and seventh postoperative day medical records of intensivists or house staffs, we could not evaluate the late complications such as cosmetic outcomes. Vertical incision tends to offer more accurate identification of anterior neck structures during PT and possibly mitigates late complications related to abnormal tracheal stoma levels. Van Heurn and colleagues [18] noted a wide variation in tracheal stoma levels in a small series of post-mortem examinations of the trachea in patients who had undergone percutaneous dilatational tracheostomy. However, tracheal stenosis and erosion into high mediastinal vessels related to a very high or very low tracheostomy site could not be observed because of the retrospective design of this study.

The limitation in this study is that patients were not randomly assigned according to incision type, as this was only a retrospective analysis. However, the optimal direction of skin incision has not been well established and the choice of a vertical versus transverse skin incision was solely dependent upon the operator’s preference. Furthermore, the percentage of experienced physicians making vertical or transverse incisions was not different in our study. However, potential selection bias resulting from operator preference was unavoidable and could affect the procedural outcomes. Another limitation is that tracheostomy was only performed in an Asian population with lower body mass index (BMI) compared to Caucasians, and the results may not be generalizable to different races. Relatively lower BMI in our study population might explain the absence of life-threatening complications after tracheostomy in our study. Last, because data were retrieved from single center, possible systemic problems intrinsic to our center may not be applicable to other centers.

## Conclusion

This retrospective study showed that transverse skin incisions in PT in critically ill patients resulted in a significant decrease in overall complications, particularly ulcers at the tracheostomy site.

## Abbreviations

APACHE II: Acute Physiology and Chronic Health Evaluation II
BMI: Body mass index
CIs: Confidence intervals
ICU: Intensive care unit
ORs: Odds ratios
PT: Percutaneous tracheostomy
ST: Surgical tracheostomy
